# Bridging the Gap between Surveillance and Interventions in Latin America Addressing Maternal and Perinatal Morbidity and Mortality

**DOI:** 10.1055/s-0043-1776732

**Published:** 2023-11-09

**Authors:** Suzanne Serruya, Maria Laura Costa, Bremen De Mucio, Pablo Duran, Claudio Sosa, Mercedes Colomar, Rodolfo Gomez Ponce de Leon, Renato Teixeira Souza, Adriana Gomes Luz, Jose Guilherme Cecatti

**Affiliations:** 1 Latin American Center for Perinatology, Women's and Reproductive Health of the Pan American Health Organization (PAHO), Montevideo, Uruguay; 2 Department of Obstetrics and Gynecology, School of Medical Sciences, University of Campinas, Campinas, SP, Brazil

The Latin American Center for Perinatology-PAHO aims to strengthen healthcare since 1970. For timely surveillance of maternal health, a perinatal information system (SIP) was implemented to enable monitoring trends of severe morbidity/mortality. It is time for integrated interventions to translate surveillance into health policies to address preventable maternal/perinatal deaths.

Regardless of the global progress in reducing maternal mortality from 2000 to 2017, the Sustainable Development Goal target is still far from the objective and if the reduction in mortality is not accelerated, Latin American countries will not meet the global or regional goals agreed. The chance of dying due to maternal causes is 10-fold higher in Latin America when compared with Europe. 1 Preventable mortality is a major concern worldwide, highlighted during the COVID-19 pandemic which resulted in a marked increase in maternal deaths. 2 Health crises expose underlying delays and disparities that need to be addressed.

How many times will we have to report an increase in adverse outcomes during crises? Recent experiences with Influenza (H1N1pdm09) and the Zika virus should have enabled a better response. Latin America has reached its turning point and needs to bridge the gap between surveillance and action to address womeńs health. It should be unacceptable to have cases of eclampsia with no treatment; preeclampsia at term, with no induction of labor; deliveries with no safe blood access, postpartum hemorrhages with no accurate interventions; delayed diagnosis and treatment of sepsis; or no access to modern contraception. Protocols and training must be implemented at all levels of reproductive, maternal and neonatal healthcare.

The Latin American Center for Perinatology, Women's Health and Reproductive Health (CLAP) of the Pan American Health Organization (PAHO) has worked to promote, strengthen and improve motheŕs and newborńs healthcare in the Region since 1970. Perinatal Information System (SIP), among accomplishments, is a free computerized clinical record system implemented in 22 countries under CLAP technical support and is a milestone in the use of systematized information. 3 SIP enables institutions to generate information to monitor the prevalence and trends of severe morbidity and mortality, quality of care, and the need for interventions for local health concerns. 4 SIP has been implemented in a network of maternities from different countries allowing institutions to surveil their own data, perform health system management, implement operational research and train human resources based on identified needs.

There are known differences among countries in the region in terms of social and economic characteristics of the population and also of available resources for maternal and child health care. In addition, there is a general agreement that existing knowledge is already sufficient for facing the challenges of prevention and treatment of causes potentially leading to severe maternal morbidity and mortality but the lack of political willingness and concrete actions to implement the required measures are the main limitations.

In conclusion, considering that CLAP/PAHO activities are feasible based on SIP soundness, as could also be on other national independent initiatives focused on the same goal of surveillance of morbidity, it is time to plan integrated interventions and translate surveillance into public health policies to effectively address preventable maternal and neonatal deaths. We urge policymakers, scientific societies and stakeholders to join forces, push toward prioritizing maternity care, and take up the available sources of information to improve womeńs lives in Latin America. This is a necessary condition if we are really willing to reach the Sustainable Development Goals on this topic by 2030.
